# Extending the novel |ρ|-based phasing algorithm to the solution of anomalous scattering substructures from SAD data of protein crystals

**DOI:** 10.1107/S2053273322008622

**Published:** 2022-10-10

**Authors:** Jordi Rius, Xavier Torrelles

**Affiliations:** a Institut de Ciència de Materials de Barcelona, ICMAB-CSIC, Campus de la UAB, Bellaterra, Catalonia 08193, Spain; Institute of Crystallography - CNR, Bari, Italy

**Keywords:** *S*_M,|ρ|_ phasing algorithm, SMAR phasing, *ipp* density modification, SAD-SMAR, |ρ|-based direct methods, structure solution

## Abstract

Owing to the importance of the single-wavelength anomalous diffraction (SAD) technique, the recently developed |ρ|-based phasing algorithm (*S*_M,|ρ|_) incorporating the inner-pixel preservation (*ipp*) procedure [Rius & Torrelles (2021). *Acta Cryst* A**77**, 339–347] has been adapted to the determination of anomalous scattering substructures and its applicability tested on a series of 12 representative experimental data sets, mostly retrieved from the Protein Data Bank. To give an idea of the suitability of the data sets, the main indicators measuring their quality are also given. The dominant anomalous scatterers are either SeMet or S atoms, or metals/clusters incorporated by soaking. The resulting SAD-adapted algorithm solves the substructures of the test protein crystals quite efficiently.

## Abstract 2

The novel *S*_M,|ρ|_ phasing algorithm has been adapted to the determination of anomalous scattering substructures from single-wavelength anomalous diffraction (SAD) data of protein crystals and successfully tested on data sets mostly retrieved from the Protein Data Bank.

## 1. Introduction

Important present applications of the single-wavelength anomalous diffraction (SAD) technique are the location of SeMet atoms in crystals of multi-site genetically engineered proteins, the determination of the positions and occupancies of the heavy atoms (or clusters) entering the crystal, *e.g.* when soaking it in a solution, or also the direct use of chemical species already present in native crystals as anomalous scatterers (S, Cl, P, …). Knowledge of the anomalous scattering (AS) substructure provides starting phase values which can be iteratively improved by density modification. Although the substructure can be solved in favourable cases by the direct interpretation of the anomalous Patterson function (Rossmann, 1961 ▸), direct methods (DM) often offer the only alternative in complex cases. The application of DM to SAD data takes advantage of the availability of the experimentally accessible absolute values of the anomalous differences (|*D*|_exp_) between pairs of acentric reflections (Bijvoet pairs) which follows from the atomic scattering factor definition

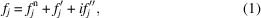

where 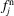 is the normal scattering factor of atom *j*, and 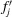 and 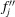 are the corresponding real and imaginary anomalous dispersion corrections (respective symbols for non-vibrating atoms are 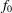 , 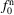 , 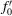 , 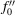 ). Let us consider a structure composed of *N* atoms with *N*_A_ of them scattering anomalously and with **r** being the atomic position vector. The structure factor of an arbitrary *H* reflection is then

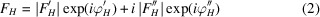

with

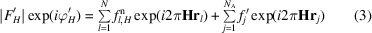




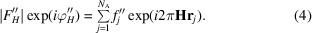




For two +*H* and −*H* reflections constituting a Bijvoet pair (from now on, 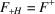 and 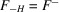 ), the absolute value of the anomalous difference *D* is given by

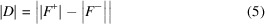

which is related to 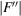 by the simple relationship (30) (see Appendix *A* [App appa])

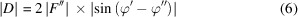

if conditions (7*a*)[Disp-formula fd7a] and (7*b*)[Disp-formula fd7b] corresponding to (28) and (29) are met, *i.e.*


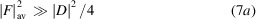

and

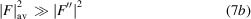

with

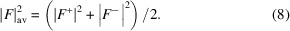




Equation (6) constitutes the basis for solving AS substructures by DM. First attempts showing the viability of locating AS in metalloproteins by DM were performed by Mukherjee *et al.* (1989 ▸) with the program *MULTAN87* (Debaerdemaeker *et al.*, 1987 ▸) following the path previously paved by Wilson (1978 ▸) in connection with the isomorphous replacement case and taking advantage of preliminary results on the location of AS using tuneable synchrotron radiation (Einspahr *et al.*, 1985 ▸); however, it was the introduction of the dual-space DM that represented a substantial improvement in the determination of AS substructures. This DM strategy refines phases by iteratively alternating structure invariant manipulation (reciprocal space) with Fourier peak optimization (real space). It was first implemented in the *Shake-and-Bake* program (Miller *et al.*, 1994 ▸). This philosophy was also incorporated in *SHELX* (Sheldrick & Gould, 1995 ▸) which evolved to *SHELXD* by incorporating, among other things, Patterson seeding (Schneider & Sheldrick, 2002 ▸). Descriptions of the application of *SHELXD* to the solution of the AS substructures are given by Usón & Sheldrick (2018 ▸) and Sheldrick (2010 ▸). More recently, the capability of SAD phasing in the presence of only weak AS has increased due to the possibility of extending the SAD experiments to longer wavelengths as well as to the availability of faster and more accurate X-ray detectors (*e.g.* Leonarski *et al.*, 2018 ▸), allowing application of lower dose rates and thus increasing data redundancy on a unique crystal (data set scaling from multiple crystals is minimized). A recent promising alternative acquisition mode, especially useful for data collection from small, weakly diffracting and radiation-sensitive crystals, is serial crystallography. This technique is based on taking one single image (containing partial Bragg reflection information) from each microcrystal and completing the diffraction data set by combining the individual indexed images from thousands of crystals. A selection of *de novo* (SAD) phasing serial crystallography studies at synchrotron sources can be found in Nass *et al.* (2020 ▸).

Recently, |ρ|-based DM in the form of the *S*_M,|ρ|_ phasing algorithm (Rius, 2020 ▸) have been extended to large crystal structures through the introduction of the peakness-enhancing *ipp* (inner-pixel preservation) procedure (Rius & Torrelles, 2021 ▸) (hereafter, to simplify its designation, the *S*_M,|ρ|_ algorithm is specified with the acronym SMAR in which S stands for ‘sum function’, M for ‘modulus function’ and AR for ‘absolute ρ’). The aim of the present contribution is the adaptation of the *ipp*-improved SMAR to the solution of AS substructures from SAD data (SAD-SMAR). Its feasibility is shown with SAD data sets either kindly supplied by the respective authors or retrieved from the Protein Data Bank (PDB). All calculations have been carried out with a modified version of *XLENS_v1* (Rius, 2011 ▸). To help the reader to assess the suitability of the test data, two indicators are given for each data set (extending to all acentric reflections in the corresponding resolution range used in the SAD-SMAR application), namely:

(i) The size of the anomalous signal (Bijvoet ratio), 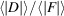 (Hendrickson & Teeter, 1981 ▸; Wang, 1985 ▸) ranging from 0.012 to 0.070 in the selected test examples.

(ii) The precision of 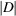 given by the 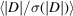 ratio (Schneider & Sheldrick, 2002 ▸; Wang, 1985 ▸) which should be >1.5 (ideally also for the outermost resolution shell) (Cianci *et al.*, 2008 ▸; Giacovazzo, 2014 ▸). Logically, the precision of 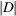 directly depends on the precision of the corresponding 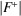 and 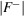 (more strictly of 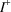 and 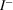 ).

In SAD phasing, redundancy of diffraction data is an important data collection parameter, since it affects the variance of the average intensity estimates. As this work is based on published data sets, the cited redundancy values are those given by the respective authors.

## 2. The composition of the |*D*| set

Solving AS substructures by DM requires a previous selection of the experimental |*D*| values, |*D*|_exp_, since not all of them are appropriate. A preliminary check should ensure that the Bijvoet-pair reflections have |*F*|_av_ values satisfying conditions (7*a*)[Disp-formula fd7a] and (7*b*)[Disp-formula fd7b]. This is accomplished by preserving in the initial set of |*D*| differences only those reflections with |*F*|_av_ values (expressed as |*E*|’s) larger than a given ECUT cut-off value. In the test calculations, the used ECUT is 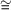 0.25 which causes the suppression of approximately 5% of the total of acentric reflections. The selection process continues with two additional rejection criteria which are directly applied to the |*D*| anomalous differences (to increase their reliability and the absence of outliers). Since |*D*| is in general much smaller than |*F*|_av_, random errors inherent to |*F* ^+^| and |*F* ^−^| seriously affect the precision of |*D*|. Consequently, only those reflections fulfilling the |*D*| > DFCUT × σ(|*D*|) criterion are preserved in the |*D*| set (Hendrickson *et al.*, 1988 ▸; Grosse-Kunstleve & Brunger, 1999 ▸). In the test calculations, DFCUT is in general ∼0.4 which represents the additional removal of 10–15% of acentric reflections from the |*D*| set. The selection process ends with the outlier elimination, *i.e.* all reflections with |*D*|/r.m.s.d.(|*D*|) greater than ∼4.0 are filtered out (Hendrickson *et al.*, 1988 ▸; Grosse-Kunstleve & Brunger, 1999 ▸) [r.m.s.d.(|*D*|) = root-mean-square deviation of |*D*|]. The surviving reflections in the |*D*| set are generically denoted by *H*.

## 3. The SAD-SMAR algorithm

### 3.1. The normalized *X* values

The SAD-SMAR algorithm uses, instead of the experimentally inaccessible quasi-normalized |*E*| values of the substructure (Main, 1976 ▸), the normalized *X* values based on (6) and defined by the quotient

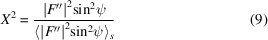

with 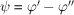 and where *s* is the resolution shell corresponding to 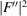 . Since 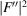 and 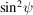 may be assumed uncorrelated, the average term in the denominator can be decomposed into the product of 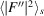 and 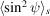 . Furthermore, since 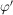 predominantly depends on the protein atoms and 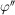 only on the anomalous scatterers, both phases can be considered largely uncorrelated and hence 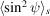 can be assumed to be 0.5, so that

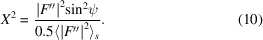




On the other hand, according to the |*E*| definition, 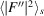 in (10) can be replaced by 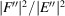 , so that the expression relating *X* ^2^ and 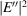 reduces to

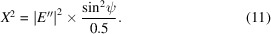




If 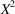 is averaged over all reflections in its corresponding *s* resolution shell, then 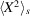 = 1, since 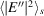 is 1 by definition and 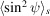 is 0.5.

In addition to *X* values, SAD-SMAR also uses modified *X* values called |*X*_m_|. These are obtained (i) by calculating the *M* modulus function with *X* as Fourier coefficients (extending the sum to the *H* reflections), (ii) by suppressing the negative regions in *M*, and (iii) by back Fourier transforming the modified *M* function (Karle, 1980 ▸).

### 3.2. Calculation of *X* from |*D*|_exp_

The relation between *X* and |*D*| is easily found by introducing the squared (6) into (10)


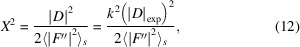

where *k* is the scaling constant putting 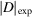 on the same scale as 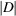 . The 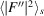 quantity in the denominator, *i.e.* the average intensity of the *s* shell, can be expressed as

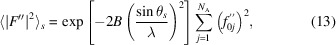

where *B* is the overall atomic displacement parameter including vibrational and disorder effects. At this point, for convenience, each 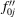 will be converted to 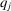 by dividing by 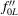 (= the largest 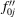 ). Replacement of 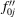 by 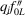 in (13) and subsequent introduction of the modified (13) into (12) leads to the final expression

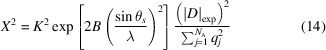

with

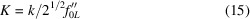

which allows the derivation of 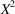 from 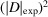 provided that the AS composition is known. In view of (14), the estimation of the *K* constant and the *B* parameter can be obtained from a Wilson plot, since for each reciprocal-space shell, both 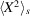 and the 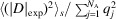 quotient are known.

### 3.3. SAD-SMAR recycling

**Figure 1:**
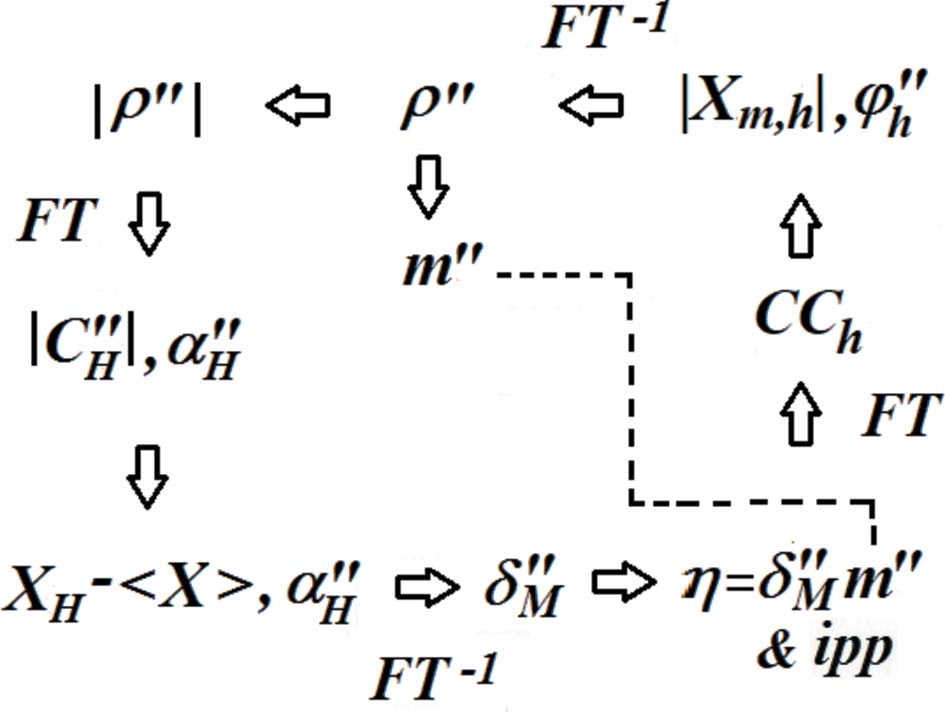
The recursive SAD-SMAR phase refinement algorithm with enhanced peakness (*ipp*). Compared with the unmodified SMAR, the principal differences are the composition of Φ_*h*_ as well as the replacement of |*E*| values either by *X* = |*E*′′sinψ| or by |*X_m_* |.

Phasing with the SMAR algorithm was first shown by Rius (2020 ▸). Later on, the *ipp* procedure, a simple way of enhancing peakness in Fourier maps, was added (Rius & Torrelles, 2021 ▸). To show how the SAD-SMAR modification works, one phase refinement cycle is described in detail in Fig. 1 ▸. It has been divided into four stages, each one including one Fourier transform operation. These are:

(i) Calculation of the 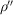 density function. The phase refinement cycle begins with the introduction of Φ_*h*_, the subset of 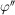 phases of the *h* reflections to be refined (either initial or updated estimates). Unlike in non-anomalous SMAR applications where Φ_*h*_ contains the phases of all large reflections (*i.e.* those *H* reflections with |*E*| ≥ 1.00), in the case of SAD-SMAR, Φ_*h*_ only includes the 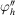 phases of those *H* reflections with *X* larger than a given XCUT cut-off (here XCUT = 1.00). Since 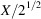 is equal to |*E*′′| |sin ψ|, the largest possible value of |*E*′′| for a given *X* is 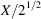 (which is reached for |sin ψ| = 1). In general, |sin ψ| will be lower than 1 and therefore 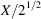 is a lower estimate of |*E*′′| (Grosse-Kunstleve & Adams, 2003 ▸). How the composition of Φ_*h*_ depends on the *X* values is illustrated in Table 1 ▸ for XCUT = 1.00. It can be seen that most phases of reflections with |*E*′′|’s > 1.00 are present in Φ_*h*_; however, this number decreases significantly for |*E*′′|’s between 1.00 and 0.70 and, finally, for |*E*′′|’s < 0.70, it becomes zero. In this work the initial estimates of 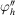 are the phase values corresponding to the Fourier coefficients of *M*′, *i.e.* the randomly shifted modulus function (Rius & Torrelles, 2021 ▸). As can be seen in Fig. 1 ▸, the Fourier synthesis with 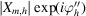 as Fourier coefficients gives the 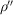 density function from which the 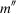 mask is derived (and stored). According to Rius (2020 ▸), 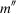 is 1 (for 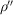 > 0), 0 (for 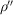 between 0 and −*t*σ) and −1 (for 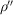 < −*t*σ) with σ^2^ being the variance of 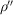 (Φ_*h*_) and *t* ∼2.65.

(ii) Calculation of the Fourier transform of | 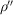 |. It gives the 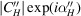 Fourier coefficients and provides the updated 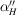 .

(iii) Calculation of 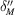 . The 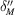 density function is the inverse Fourier transform of the 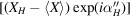 coefficients formed by the experimental 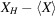 values and the updated 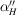 phases. The calculated 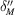 is then multiplied with the previously stored 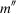 mask to give the η product function.

(iv) Calculation of the Fourier transform of η. Peakness in η is enhanced by applying the *ipp* density modification procedure. Once completed, the modified η is Fourier-transformed to provide the new 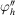 and 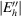 values, the latter being used in the calculation of the 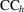 figure-of-merit to follow the phase refinement convergence,

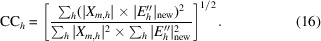

If convergence is not achieved, the next cycle begins until the preset maximum number of cycles is reached.

## 4. Fourier refinement and figure-of-merit

After applying SAD-SMAR, the phases are further refined by Fourier recycling (five to ten cycles). In order not to have to modify the Fourier refinement module of already existing DM programs, *e.g.* of *XLENS_v1* (Rius, 2011 ▸), the 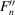 structure factor corresponding to a hypothetical structure with scatterers of 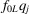 strengths is introduced (with 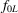 being the normal scattering factor corresponding to the largest 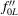 ). For this purpose, (11) and (14) are equated and both sides of the expression multiplied by 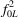 . After rearranging the resulting expression, we obtain

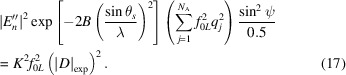




Notice that the first three factors of the left-hand side of (17) correspond to 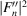 . Replacement of these by 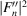 gives, after taking the square root, the best approximation Γ to the modulus of the structure factor

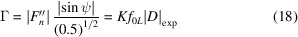

which is used as observational data in the 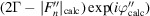 Fourier coefficients during recycling. At the end of the last Fourier refinement cycle, the (correlation coefficient based) residual is calculated

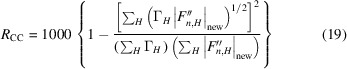

wherein the sums only include the *H* reflections with *X* ≥ 0.7.

## 5. Results of the test calculations

Relevant experimental information about the data sets used in the test calculations is given in Table 2 ▸. To improve the readability of the text, the test compounds are simply referenced with the appropriate PDB code. The verification of the SAD-SMAR tests was greatly facilitated by the availability of the refined model coordinates either kindly provided by the authors or deposited by them in the PDB. In this way, the r.m.s.d.’s between our substructure models and the deposited ones could be calculated. The most relevant results of the test calculations are summarized in Table 3 ▸. Table 4 ▸ complements this information by giving, for most test examples, the peak heights at the end of the Fourier recycling stage. Peak heights are always given in ρ_peak_/σ units, where ρ_peak_ is the density at the peak centre and σ^2^ is the variance of ρ.

To get a rough idea of the quality of the deposited/supplied SAD refinements, the deposited *R*_free_ values (listed in Table 2 ▸ together with the corresponding upper resolution limits, RES_ref_) were compared with the median *R*_free_ values of the PDB which are 0.24, 0.25, 0.26 and 0.28 for upper resolution limits corresponding to the intervals 1.95–2.15, 2.15–2.35, 2.35–2.40 and ∼2.90 Å (Read *et al.*, 2011 ▸). It is found that the *R*_free_ values are less than or equal to the corresponding median *R*_free_ values in all cases, except for 2g4s and 4tno, for which *R*_free_ is significantly higher.

A preliminary test was the substructure solution of the proenzyme of proabylysin (PDB code 4jiu; *a* = 34.679, *b* = 44.896, *c* = 72.233 Å, *P*2_1_2_1_2_1_). The data set was measured at ID29 (ESRF) at the Zn absorption edge (λ = 1.282 Å) (López-Pelegrin *et al.*, 2013 ▸). The structure refinement (deposited by the same authors) contains one Zn ion, one macromolecule and 148 water molecules in the asymmetric unit (a.u.), amounting to 1055 atoms. The successful run of this simple case (separation between found and deposited Zn ion positions is ∼0.15 Å) confirmed the capability of SAD-SMAR to solve AS substructures at 2.5 Å resolution (*B* 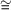 25.1 Å^2^). Next, it was tested with more challenging cases. To simplify the discussion, the test compounds are divided into three groups.

### 5.1. SeMet derivatives

Compared with other SAD situations, Se-SAD is particularly favourable due to the large AS strength of Se ( 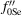 ∼3.9 and ∼3.3 e^−^ for λ = 0.979 and 0.919 Å, respectively) and because the substitution of S by Se in the me­thio­nine amino acids is normally complete. The data sets of the three tested SeMet derivatives correspond to:

5cx8: *a* = 56.64, *b* = 184.74, *c* = 144.31 Å, *P*2_1_2_1_2. A major immunodominant outer-membrane surface receptor antigen of *Porphyromonas gingivalis* measured at beamline (BL) XALOC (ALBA, Barcelona) (Goulas *et al.*, 2016 ▸; Se derivative refinement deposited in PDB entry 5cx8; SAD data supplied by one of them). There are 12 Se positions, two macromolecules and 509 water molecules in the a.u., amounting to 8119 atoms. Application of SAD-SMAR yields the positions of the 12 Se atoms (*B* 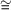 2.3 Å^2^) with r.m.s.d. = 0.24 Å compared with the deposited refined model (Table 3 ▸).

4yu5: *a* = 97.61, *b* = 102.41, *c* = 242.88 Å, *P*2_1_2_1_2_1_. Thuringilysin, a variant of zymogenic BaInhA2-E/A measured at BL XALOC (ALBA, Barcelona) (Arolas *et al.*, 2016 ▸; Se derivative refinement deposited in PDB entry 4yu5; SAD data supplied by one of them). There are 18 Se, one Zn, two macromolecules and 104 water molecules in the a.u., amounting to 10 942 atoms. Application of SAD-SMAR supplies the positions of the 18 Se atoms (*B* 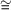 9.8 Å^2^) with r.m.s.d. = 0.35 Å. Regarding the Zn ion, it shows up in the Fourier map 1.06 Å apart from the deposited refined position. Its strength is similar to that of the two Se atoms with higher *B* values.

5lac: *a* = 94.144, *b* = 111.353, *c* = 58.191 Å, *P*2_1_2_1_2. A 3C-like protease of Cavalli virus collected at BL 14.2 (BESSY II, Berlin) (Kanitz *et al.*, 2019 ▸; SAD and refinement data deposited in PDB entry 5lac). There are 12 Se positions (one of them split in the refinement), one macromolecule and 303 water molecules in the a.u., amounting to 4875 atoms. Application of SAD-SMAR yields the positions of the 12 Se atoms (*B* 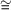 4.9 Å^2^) with r.m.s.d. = 0.18 Å compared with the deposited model.

### 5.2. Native crystals soaked in heavy metal/metal cluster solutions

The first four cases of this subsection are native crystals soaked in a solution containing iodide ions and with their diffraction data being collected in-house on rotating anodes (Cu *K*α radiation) where the anomalous signal for I is large ( 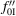 ∼6.9 e^−^). The fifth case corresponds to crystals soaked in a Cd^2+^-containing solution.

**Figure 2:**
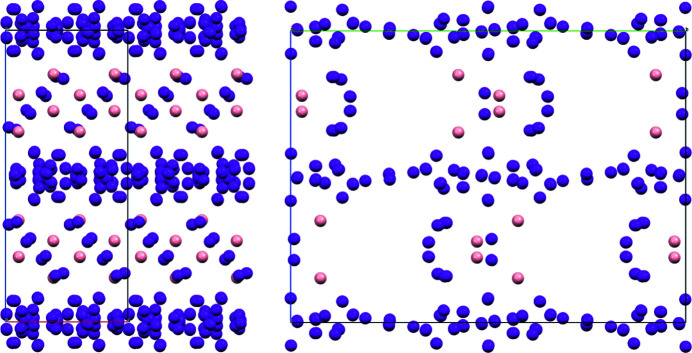
5iqy: (010) and (100) projections of the I^1−^ site arrangement in the unit cell (only sites with occupancies ≥ 0.40): (violet) 120 (= 15 × 8) sites obtained by SAD-SMAR and Fourier recycling (which are also present in the deposited refinement; r.m.s.d. between found and deposited sites is 0.43 Å); (pink) 16 (= 2 × 8) additional sites only present in the deposited refinement (0.53 and 0.40 occupancies) (see Table 5 ▸).

5iqy: *a* = 40.89, *b* = 132.08, *c* = 97.57 Å, *C*222_1_. An apo-de­hydro­ascorbate reductase from *Pennisetum glaucum *(Krishna Das *et al.*, 2016 ▸; SAD and refinement data deposited in PDB entry 5iqy). According to the deposited data, there are 26 sites occupied by a total of 13.3 I^1−^, one macromolecule and 95 water molecules in the a.u. (1719 atoms). Application of SAD-SMAR yields 15 sites (*B* 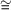 45.5 Å^2^) containing 9.74 I^1−^ which show a good agreement with the deposited data (r.m.s.d. = 0.43 Å) as shown in Fig. 2 ▸. Table 5 ▸ compares the resulting site occupancies with the deposited ones.

3k9g: *a* = 55.81, *c* = 200.90 Å, *P*4_3_12. A plasmid partition protein (Abendroth *et al.*, 2011 ▸; SAD and refinement data deposited in PDB entry 3k9g). According to the structure refinement deposited in the PDB, there are 12 I^1−^ sites, one macromolecule and 91 water molecules in the a.u. (1858 atoms) with 6.6 I^1−^ in the 12 sites. Application of SAD-SMAR yields nine coincident I^1−^ sites (*B* 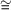 19.8 Å^2^) (r.m.s.d. = 0.35 Å) which justify a total of 5.3 I^1−^, *i.e.* 81% of the refined I^1−^content. By normalizing the sum of the heights of the nine strongest Fourier peaks to 5.3, the respective found and deposited site occupancies (using the original site labelling) are I1: 0.91, 0.99; I2: 0.91, 1.00*; I3: 0.60, 0.61; I4: 0.50, 0.53; I5: 0.59, 0.38; I6: 0.54, 0.38; I7: 0.60, 0.53; I10: 0.32, 0.36; I12: 0.33, 0.29 (* truncated to 1.00).

3km3: *a* = 84.66, *c* = 140.74 Å, *R*3(H). A de­oxy­cytidine triphosphate deaminase (Abendroth *et al.*, 2011 ▸; SAD and refinement data deposited in PDB entry 3km3). The refinement in the PDB includes 16 I^1−^ sites, two macromolecules and 516 water molecules in the a.u. (10 752 atoms) with 10.9 I^1−^ in the 16 sites. SAD-SMAR gives 13 coincident I^1−^ sites (*B* 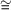 6.2 Å^2^) (r.m.s.d. = 0.43 Å) which justify the 87% of the refined I^1−^content. (Due to the large variability of the individual isotropic *B* values affecting the metal sites, no attempt to estimate the site occupancies from the corresponding peak heights was made.)

3men: *a* = 45.70, *b* = 162.12, *c* = 173.07 Å, *P*2_1_2_1_2_1._ An acetyl­polyamine amino­hydro­lase (Abendroth *et al.*, 2011 ▸; SAD and refinement data deposited in PDB entry 3men). According to the deposited data, there are 35 sites occupied by ∼23.1 I^1−^, four macromolecules and 516 water molecules in the a.u. (10 825 atoms). Application of SAD-SMAR yields 33 coincident I^1−^ sites (*B* 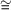 10.5 Å^2^) (r.m.s.d. = 0.24 Å) which justify ∼92% of the refined I^1−^content. The r.m.s.d. between the 33 found and corresponding deposited site occupancies is 0.172.

2g4h: *a* = 182.16 Å, *F*432. A Cd-containing apoferritin measured at BL X12 (EMBL/DESY, Hamburg) (Mueller-Dieckmann *et al.*, 2007 ▸; SAD and refinement data deposited in PDB entry 2g4h). Anomalous signal for Cd^2+^ at λ = 2.00 Å is large ( 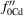 ∼7.2 e^−^). According to the deposited refinement, the a.u. contains five Cd^2+^ sites (with occupancies > 0.10), two Cl^1−^ sites, 101 water molecules and one apoferritin subunit (a macromolecule with 1374 atoms). Apoferritin is made up of 24 such protein subunits which assemble to form a roughly spherical hollow shell, with an external diameter of ∼120 Å and an internal diameter of ∼80 Å (Chrichton, 2019 ▸). The shell is placed at the nodes of the *F* lattice complex. Application of SAD-SMAR yields the five Cd^2+^ sites (*B* 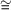 33.7 Å^2^) with the found positions and occupancies close to the deposited values (r.m.s.d. between corresponding sites is 0.32 Å). The respective found and deposited occupancies (using the original site labelling) are Cd1: 0.50, 0.50; Cd2: 0.25, 0.25; Cd3: 0.14, 0.20; Cd4: 0.20, 0.18; Cd5: 0.14, 0.16). The Cd1 sites are located pairwise (∼8 Å separation) at the 12 vertices of a cubo-octahedron centred at (0, 0, 0) (with opposite vertices separated by ∼129 Å), *i.e.* close to the external diameter of the hollow shell. The same applies for Cd2 but with a somewhat longer intra-pair distance (∼13 Å) and a separation between opposite vertices of ∼75 Å which roughly corresponds to the internal diameter of the hollow shell.

### 5.3. S-SAD phasing

The data sets of Pf1117 and Pf0907, two hypothetical proteins from *Pyrococcus furiosus*, were collected at BL X06DA at the Swiss Light Source (Weinert *et al.*, 2015 ▸; the corresponding SAD and refinement information deposited with respective PDB codes 4tno and 4pgo).

4tno: *a* = 47.21, *c* = 82.28 Å; *P*4_1_2_1_2. According to the deposited data, its a.u. contains one macromolecule, three me­thio­nine S atoms and two Cl^1−^ (709 atoms; 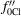 ∼ 1.20 and 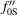 ∼ 0.95 e^−^). Application of SAD-SMAR yields the two Cl^1−^ and two S atoms (*B* 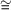 47.9 Å^2^). The third (more disordered) S atom could not be located. The r.m.s.d. between found and deposited positions is 0.48 Å.

4pgo: *a* = 88.50, *c* = 73.12 Å; *P*6_5_22. The deposited data indicate that besides the macromolecule and water molecules, there are two me­thio­nine S atoms and two Cl^1−^ in the a.u. (∼689 atoms). Application of SAD-SMAR leads to the same AS model (*B* 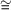 57.3 Å^2^) with r.m.s.d. = 0.56 Å.

The third and last example is the PB1 domain of the human scaffold protein NBR1 (Müller *et al.*, 2006 ▸):

2g4s: *a* = 101.40, *c* = 42.59 Å; *P*6_3_22. The data set was collected at BL X12 (EMBL/DESY, Hamburg) (Mueller-Dieckmann *et al.*, 2007 ▸; SAD and refinement data deposited in PDB entry 2g4s). According to the deposited refinement, the a.u. contains, besides the macromolecule and the refined water molecules, four me­thio­nine S atoms (one of them with a higher *B* value) (689 atoms; 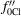 ∼1.11 and 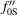 ∼0.91 e^−^). Application of SAD-SMAR shows the four expected S atoms (*B* 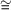 42.6 Å^2^), three of them as the three strongest Fourier peaks with a r.m.s.d. of only 0.18 Å compared with the deposited model. The fifth-ranked Fourier peak corresponds to the fourth S atom (the one with the higher *B* value in the refinement) and is shifted by 1.1 Å from the deposited position. The fourth-ranked Fourier peak could not be assigned (perhaps corresponding to some missing Cl^1−^).

## 6. Conclusions

Based on the experimental conditions covered by the test examples, it may be concluded that SAD-SMAR can solve efficiently AS substructures from SAD data (i) with upper resolution limits (RES_SMAR_) between 2.50 and 3.3 Å; (ii) with average Bijvoet ratios of 0.065 (for SeMet derivatives), 0.014 (for S-SAD phasing) and 0.041 (for soaked native crystals); (iii) with 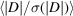 values greater than 1.5; and (iv) with 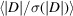 values for the outermost resolution shell ranging from 0.90 to 1.69 (the average being 1.25). The cut-off values of the various rejection criteria used in the tests have been ECUT 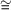 0.25, r.s.m.d.(|*D*|) = 4 and DFCUT = ∼0.4. The introduction of DFCUT ensures the suppression of the less reliable |*D*|’s while keeping enough observations for a satisfactory DM run. It can be clearly seen that the corresponding CC_*h*_ values are close to 0.88 for converging trials (with the corresponding *R*_CC_ values lying between 51 and 69). Since for non-converging trials CC_*h*_ values are normally smaller by 0.02–0.03 (and *R*_CC_ values are in general 1.3–1.4 times larger), identification of the correct trials should not be a problem. Notable is how quickly convergence is reached, especially for SeMet derivatives and for soaked native crystals. For native crystals with only S and/or Cl as AS, the test results clearly indicate that SAD-SMAR can be successfully applied to them. In the three test structures, the S atoms belong to me­thio­nine amino acids and no disulfide bridges are present. Since SAD-SMAR only considers the lattice symmetry operations, it processes the initial phase estimates derived from the randomly shifted *M*′ modulus function quite efficiently (Rius & Torrelles, 2021 ▸). As shown in Table 4 ▸, the ρ_peak_/σ limit for considering the peaks at the end of Fourier recycling as part of the structure model can usually be set between 5.0 and 7.0.

To evaluate the influence of the DFCUT value in the phase refinement results, the test calculations were repeated with DFCUT = 0.0 and the results included in Table 3 ▸ for comparison. It can be seen that, for converging trials, the CC_*h*_ values are similar (∼0.88) and the *R*_CC_ values are 2 or 3 units larger (an increase which is otherwise logical since the less reliable |*D*| values enter in the calculation). The comparison of the number of converging trials (n.c.t.) for both series of calculations indicates that DFCUT = ∼0.4 gives significantly higher n.c.t. values only for 2g4s and 4tno (by factors 1.89 and 1.10, respectively). This is surely related to their higher *R*_free_ values (0.323 and 0.305, respectively) when compared with the median *R*_free_ value of the PDB (0.265).

One characteristic of SAD-SMAR is the delivery of almost complete models when it converges. Most probable causes of non-convergence are, besides the poor quality of the experimental data, some functional limitations of the model description, *e.g.* when the resolution of the data is not enough to resolve the AS peaks in the Fourier map. Fortunately, due to the large separation among anomalous scatterers, this limitation is generally not a problem. However, at intermediate resolutions (>2.0 Å), the presence of disulfide bridges in proteins, *e.g.* between cysteine residues, represents a limitation of the otherwise highly effective *ipp* procedure (the approximate spherical symmetry of individual S Fourier peaks is lost in the overlapped S–S peak). This problem has already been addressed in *SHELXD* (Usón & Sheldrick, 2018 ▸; Sheldrick, 2010 ▸). It is clear that adapting the *ipp* philosophy to the treatment of disulfide bridges would considerably expand the scope of SAD-SMAR in S-SAD phasing.

## Supplementary Material

Output of SAD_SMAR solutions, and comparison of SAD_SMAR efficiency for DFCUT=0.4 and DFCUT=0.000. DOI: 10.1107/S2053273322008622/ae5119sup1.zip

## Figures and Tables

**Table 1:** Effect of XCUT on the composition of the Φ_*h*_ subset of phases The central part of the table lists the |*E*′′|| sin ψ | products for selected |*E*′′| and |sin ψ| values (numbers in bold refer to XCUT = 1.00). As shown in the rightmost column, Φ_*h*_ contains no phases of reflections with |*E*′′| < 0.70; however, for |*E*′′| > 1.00, the percentage of reflections considered in Φ_*h*_ is very high, *e.g.* 85.56% for |*E*′′| = 2.

	| sin ψ |					
|*E*′′|	1.00	0.75	0.50	0.25	0.10	% in Φ_*h*_
3.00	**3.00**	**2.25**	**1.50**	**0.75**	0.30	94.28
2.00	**2.00**	**1.50**	**1.00**	0.50	0.20	85.56
1.00	**1.00**	**0.75**	0.50	0.25	0.10	55.56
0.71	**0.71**	0.53	0.36	0.18	0.07	6.40
0.50	0.50	0.38	0.25	0.13	0.05	0.00
0.10	0.10	0.08	0.05	0.03	0.01	0.00

**Table 2:** Relevant data collection parameters and indicators ^1^Detailed author references in Section 5; ^2^main anomalous scatterers; ^3^redundancy of diffraction data taken from the published/deposited data (and later normalized to the point group order); ^4^highest resolution (in Å) of SAD data used in the structure refinement with *R*_free_ values^5^ from the respective authors; ^6^highest resolution for SAD-SMAR application; Bijvoet ratio^7^ estimation; and 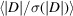 ^8^ calculations (in the whole range and in the outermost reciprocal-space shell).

PDB code^1^	AS^2^	Space group	λ (Å)	Redundancy^3^	RES^4^_ref_	*R*_free_^5^	RES^6^_SMAR_	〈|*D*|〉^7^/〈|*F*|〉	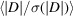 ^8^	
									Whole	Outer
4jiu^(*a*)^	Zn	*P*2_1_2_1_2_1_	1.282	1.63	1.60	–	2.50	0.0452	1.41	1.36
5cx8^(*b*)^	Se	*P*2_1_2_1_2	0.979	1.68	2.40	0.208	3.00	0.0568	1.59	0.80
4yu5^(*c*)^	Se	*P*2_1_2_1_2_1_	0.979	1.10	2.90	0.207	3.30	0.0693	1.57	0.90
5lac^(*d*)^	Se	*P*2_1_2_1_2	0.918	1.15	1.94	0.207	2.50	0.0696	2.51	1.60
5iqy^(*e*)^	I	*C*222_1_	1.542	6.83	2.40	0.234	3.00	0.0624	3.13	1.69
3k9g^(*f*)^	I	*P*4_3_2_1_2	1.542	1.56	2.25	0.266	2.90	0.0433	2.68	1.41
3km3^(*f*)^	I	*R*3(H)	1.542	1.87	2.10	0.222	2.80	0.0361	1.60	1.01
3men^(*f*)^	I	*P*2_1_2_1_2_1_	1.542	1.70	2.20	0.237	3.00	0.0466	1.71	1.06
2g4h^(*g*)^	Cd	*F*432	2.000	3.04	2.00	0.218	2.90	0.0141	3.71	1.46
4tno^(*h*)^	S, Cl	*P*4_1_2_1_2	2.066	9.53	2.14	0.305	2.60	0.0132	2.37	1.12
4pgo^(*h*)^	S, Cl	*P*6_5_22	2.066	8.58	2.30	0.203	3.00	0.0175	3.03	1.21
2g4s^(*g*)^	S	*P*6_3_22	2.000	2.86	2.15	0.323	3.20	0.0116	1.93	1.47

(*a*) López-Pelegrín *et al.* (2013 ▸); (*b*) Goulas *et al.* (2016 ▸); (*c*) Arolas *et al.* (2016 ▸); (*d*) Kanitz *et al.* (2019 ▸); (*e*) Krishna Das *et al.* (2016 ▸); (*f*) Abendroth *et al.* (2011 ▸); (*g*) Mueller-Dieckmann *et al.* (2007 ▸); (*h*) Weinert *et al.* (2013 ▸).

**Table 3:** Comparison of the SAD-SMAR phase refinement results for DFCUT = ∼0.4 and 0.0 ^1^Completeness as *c_D_* = *N_D_*/*N*_asy_ in % (*N*_*D*_ = number of reflections in |*D*| set; *N*_asy_ = number of unique reflections); ^2^n.c.t. = number of converging (correct) trials out of 25; ^3^(average) number of cycles to reach convergence; ^4,5^final CC_*h*_ and *R*_CC_ values for correct solutions; ^6^number of sites found in the a.u. compared with published refined values; ^7^sep. = root-mean-square deviation in Å between found and published refined site positions.

PDB code	DFCUT	*c_D_* ^1^	n.c.t.^2^	ncycle^3^	CC_*h*_^4^	*R*_CC_^5^	nsites^6^	Sep.^7^
4jiu	0.375	71.2	25	5	0.91	39	1/1 Zn	0.15
	0.0	85.4	25	5	0.91	41		
5cx8	0.375	71.6	25	12	0.88	59–61	12/12 Se	0.24
	0.0	86.6	25	10	0.88	62–64		
4yu5	0.375	71.0	25	13	0.87	60–62	18/18 Se	0.35
	0.0	87.2	25	14	0.85	68–69		
5lac	0.375	87.6	25	27	0.91	50–51	12/12 Se	0.18
	0.0	90.1	25	14	0.91	49		
5iqy	0.450	76.2	8	<50	0.87	57–59	15/26 I	0.43
	0.0	86.0	13	<50	0.87	54–61		
3k9g	0.375	73.8	21	<55	0.87	59–65	9/12 I	0.35
	0.0	81.8	20	<55	0.87	60–64		
3km3	0.375	78.2	18	<36	0.87	65–69	13/16 I	0.43
	0.0	93.1	24	<45	0.87	68–71		
3men	0.375	74.1	4	<100	0.88	55–58	33/35 I	0.24
	0.0	88.0	11	<55	0.88	57–58		
2g4h	0.750	68.1	25	<50	0.87	57–61	5/5 Cd	0.22
		80.8	25	<50	0.87	59–63		
4tno	0.400	67.3	22	<30	0.88	51–54	4/3 S + 2 Cl	0.48
	0.0	79.2	20	<30	0.88	53–56		
4pgo	0.375	70.9	22	<40	0.88	54–57	4/2 S + 2 Cl	0.56
	0.0	79.9	25	<40	0.87	55–59		
2g4s	0.375	67.6	19	<125	0.88	57–60	3/4 S	0.18
	0.0	79.3	10	<125	0.88	59–62		

**Table 4:** Heights of peaks in the final map of Fourier recycling for most test examples expressed in ρ_peak_/σ units (ρ_peak_ = maximum peak density; σ^2^ = variance of ρ) The peaks in the a.u., ordered in decreasing height, are divided into two sets: *A* containing all correct signal peaks down to the first uninterpreted peak (only the heights of the first and last peaks are given, followed by the corresponding number of AS in brackets); *B* with mixed correct and uninterpreted peaks (with the heights of the latter in italics). According to these results, cut-off values of ρ_peak_/σ(ρ) for considering Fourier peaks as part of the substructure model can be set at around 5.0–7.0 (for soaked native crystals, they are slightly higher).

Code	*A*	*B*
5cx8	43.2 → 20.6 [12 Se]	*5.6*
4yu5	21.1 → 16.3 [18 Se +1 Zn]	15.0, *12.5*, *7.0*
5lac	48.0 → 12.4 [12 Se]	*5.5*
5iqy	17.8 → 7.0 [14 I]	*6.8*, *6.6*, 6.3, 5.3, *5.3*, 5.1, *5.0*
3k9g	35.4 → 10.8 [8 I]	*9.9*, 9.2, *8.8*
3km3	35.6 → 14.4 [9 I]	*13.5*, 12.5, 10.9, 10.7, *7.4*, 7.2, *7.1*
3men	36.3 → 8.7 [32 I]	*8.1*, 8.0, *7.6*
4tno	17.0 → 11.0 [2 S, 2 Cl]	*5.3*
4pgo	23.9 → 9.6 [2S, 1 Cl]	*8.1*, 6.4, *6.3*
2g4s	17.2 → 14.0 [3 S]	*6.4*, 5.9, *5.6*

**Table 5:** 5iqy: list of top-ranked iodide site occupancies (≥0.40) obtained by applying the SAD-SMAR algorithm compared with those in the deposited refinement (Krishna Das *et al.*, 2016 ▸) (see Fig. 2 ▸) Only two peaks are missing. (Sep = separation between corresponding sites.)

Site No.	Occ. SAD-SMAR	Occ. LS	Sep. (Å)
1	1.00	1.00	0.11
2	0.90	0.76	0.12
3	0.82	0.86	0.14
4	0.76	0.78	0.57
5	0.72	0.62	0.34
6	0.71	0.65	0.25
7	0.67	0.62	0.52
8	0.62	0.66	0.34
9	0.60	0.63	0.45
10	–	0.53	–
11	0.48	0.44	0.81
12	0.48	0.60	0.46
13	0.44	0.54	0.24
14	0.40	0.40	0.36
15	0.40	0.42	0.55
16	–	0.40	–
17	0.40	0.40	0.55

## Appendix A

By considering  in expression (2), this can be written as

Multiplication of  and  by their respective complex conjugates gives, after some algebraic manipulation (Ramachandran & Srinivasan, 1970 ▸),

Addition of  and  leads to

Likewise, by calculating their difference, the expression

is obtained which when squared yields

wherein

By squaring (26), it follows, after rearranging terms, that   . Replacement of  in (25) gives

Finally, for Bijvoet pairs satisfying the conditions

the fractional term in (27) tends to 1, so that

holds.

This expression is also valid for structures containing anomalous scatterers of different type.
